# How the Doctrine of Double Effect Rhetoric Harms Patients Seeking Voluntary Assisted Dying

**DOI:** 10.1007/s11673-024-10340-4

**Published:** 2024-03-29

**Authors:** E. Kendal

**Affiliations:** Department of Health Sciences and Biostatistics, Swinburne University of Technology, John St, Hawthorn, VIC 3122 Australia

**Keywords:** Voluntary assisted dying, Euthanasia, Doctrine of double effect, Abortion

## Abstract

Victoria’s Voluntary Assisted Dying Act 2017 (Vic) became the first state law to permit VAD in Australia under limited circumstances from June 2019. Before this, many palliative care physicians relied on the doctrine of double effect (DDE) to justify the use of pain relievers for terminally ill patients that were known to hasten death. The DDE claims that there is a morally significant difference between intending evil and merely foreseeing some bad side-effect will occur as a result of one’s actions. This article argues that the legacy of the DDE is promoting inequitable access to VAD in Victoria due to the assumption that death represents an “evil” for the patient and that the intentions of physicians providing VAD cannot be trusted. The latter claim relies on two common objections to the DDE: the risk of “purifying the intentions” and the issue of “closeness” when evaluating moral acts under this theory.

Until recently, voluntary assisted dying (VAD) was illegal in Australia, with Victoria’s *Voluntary Assisted Dying Act 2017 (Vic)* becoming the first state law to permit VAD under limited circumstances from June 2019 (Victorian Government 2021, henceforth VAD Act). Before this, many palliative care physicians relied on the doctrine of double effect (DDE) to justify the use of pain relievers for terminally ill patients that were known to hasten death. The DDE claims that there is a morally significant difference between *intending* evil as a means to a certain end and merely *foreseeing* some bad side-effect will occur as a result of one’s actions (Hull, 2000, 195). Put simply, as long as the physician did not *intend* to euthanize the patient, their method of achieving the morally permissible goal of relieving their patient’s suffering was considered legal and ethical, even if it ultimately brought about the death of the patient as a foreseeable but unavoidable consequence. In this article, I argue that the legacy of the DDE is promoting inequitable access to VAD in Victoria due to the inherent assumption that death represents an “evil” for the patient that they must be protected from and that the intentions of physicians providing VAD cannot be trusted. The latter claim relies on two common objections to the DDE: the risk it allows agents to justify any action so long as they can “purify” their intentions and the issue of “closeness” when evaluating moral acts under this theory.

## 1: Introducing the Doctrine of Double Effect

According to Raanan Gillon (1986), the DDE was first developed by Thomas Aquinas in an attempt to “delineate the conditions in which it is morally legitimate to cause or permit evil in the pursuit of good” (193). The doctrine dictates that when an action can reasonably be expected to produce both good and bad outcomes, the agent’s intention is the deciding factor in determining whether or not pursuing the action is acceptable. Where the intention is to act to bring about the good outcome, merely foreseeing that the bad outcome is also likely to be brought about as a side-effect, the DDE states the action is permissible. Alternatively, where the intention is to cause the bad outcome, or the good outcome is a *direct result* of the bad outcome, the DDE states the action is impermissible. Thus, there are four criteria in the DDE that an action must satisfy to be considered acceptable:

1. The act when considered independent of its consequences must be of the sort that is morally permissible
2. The intended outcome must be good, while the bad outcome can be foreseen but never intended
3. The bad outcome must not be the method of obtaining the good outcome, i.e. it must not be the *means* of achieving the desired goal
4. The benefit of the good outcome must reasonably outweigh the harm of the bad outcome (Kendall 2000, 204).

When used in medicine, where the same medical intervention can be seen to bring about both positive and negative effects, the moral defensibility of pursuing such a treatment depends on which outcome the physician *intends* when administering it. The DDE does not defend any treatment in which the negative effect is the *cause* of the positive result but only when the bad outcome is a foreseeable side-effect of the action intended to bring about the good outcome. According to Richard Hull (2000), the applicability of the DDE therefore rests on the possibility of distinguishing “bad means and means that also produce the bad” (196). But herein lies the problem. By relying on the rhetoric of “bad” side-effects in historical discussions of euthanasia and the DDE, we effectively neglect the possibility that death can be characterized as a positive and desirable outcome for those patients seeking VAD. We also reject the reality that intending to cause death as a *means* of alleviating suffering may be ethically sound. I argue it is these prejudices and assumptions that have led to our overly restrictive criteria for accessing VAD in Victoria.

## 2: The Benefit of the Doctrine of Double Effect for Moral Guidance

One of the major benefits of adopting the DDE into practical ethics must surely be the special place this doctrine affords intention, unlike many consequentialist moral theories (Quinn 1989, 334). According to Neil Francis Delaney (2007), this aspect provides the DDE “a perfectly secure place not only in philosophical discourse but in ordinary commonsense,” since it is widely accepted that when an action can have more than one effect it is *the thought that counts* (106). Gillon (1986) claims this signifies a “widespread and deeply held intuition” among the rational members of society, that intention is a relevant consideration when evaluating the moral behaviour of an agent (193). Thus, the DDE allows for commonly held beliefs regarding the difference between bringing about a good outcome for the right reasons, and intending an evil means or end, regardless of whether the actual consequences of the action are the same.

While the classical examples used to teach DDE both involve “killing the innocent,” (either through opiate-based pain relief for the terminally ill or a coincidental termination of pregnancy when performing a hysterectomy), I argue there is a better case for evaluating the legitimacy of this theory for determining the role of intention in moral judgments. Let us consider a scenario in which the DDE can be called upon to justify the actions of a physician providing inoculations to children, despite knowing the pain and distress this will likely cause. Using the DDE, we would intuitively distinguish between the morality of one doctor who, with the intention of preventing disease, chooses to expose children to the distress of a painful injection, compared to a second doctor who brings about the same level of immunity but does so with the intent of inflicting pain on children. Returning to the four criteria established in the DDE, in the first instance, assuming vaccinating children is a morally permissible act in itself, the actions of the doctor here are acceptable, since it is the good outcome (immunity) that is the intended outcome and the bad outcome (pain and distress) is merely a foreseen side-effect. It is the vaccine that is the means of achieving the desired outcome, and not the pain of the injection. Thus, the bad outcome is not the *means* of producing the good outcome but rather is an unfortunate by-product of the necessary means of injecting the vaccine into the bloodstream. On the other hand, the second doctor, who administers vaccinations with the intention of causing pain and torturing children, can reasonably be labelled as acting wrongly, despite engaging in the exact same activity as the first doctor. The mere consequence of producing immunity is not sufficient in determining whether each doctor behaves morally, as both achieve this effect, but rather it is the *intention* with which each acts that is useful in evaluating their moral stature.

The above example also has the advantage of allowing for investigation of the DDE in both individual and population-based outcomes, which neither the end-of-life pain relief nor the therapeutically indicated abortion examples can provide. This is because the doctor giving the injections can also be expected to predict a small percentage of adverse reactions among a large group, as well as foreseeing the pain and distress of each individual child. However, even when introducing a more serious potential side-effect into the vaccination example, the first doctor can still be said to be behaving morally, so long as their intention is not to bring about these negative reactions. This is also relevant for our discussion of VAD, as some of the restrictions placed on accessing this intervention seem to rely on concerns for potential third-party harm: e.g., are based on a belief that making VAD difficult to access at the individual level protects the public interest.

## 3: Accessing Voluntary Assisted Dying in Victoria: Analyzing the Legislation

The eligibility criteria for VAD in Victoria are among the strictest in the world. To qualify, a patient must be over eighteen, an Australian citizen or permanent resident, a Victorian resident (and have resided in Victoria for at least twelve months at the time of the initial request), be judged to have decision-making capacity regarding VAD, and have a terminal diagnosis of an incurable, advanced and progressive “illness or medical condition” that is expected to cause death within six months (twelve months for neurodegenerative conditions), and which is causing irremediable and intolerable suffering (VAD Act, Part 2, subsection 9:1). Patients who are diagnosed with a mental health condition or disability under the *Mental Health Act 2014* or *Disability Act 2006* are not eligible unless they also possess a diagnosis that fits the above description (e.g., someone with a spinal cord injury would not qualify unless they also had, for example, a terminal cancer prognosis indicating death was expected within six months) (VAD Act, Part 2, subsections 9:2; 9:3). The process itself is also quite onerous, requiring a first request, first assessment, consulting assessment, signed and witnessed written declaration, a final request, and a final review. In addition to the person requesting access, the process involves a coordinating medical practitioner, a consulting medical practitioner, relevant specialists (if a referral for expert opinion is required), a dispensing pharmacist and a contact person who is later responsible for returning any unused VAD substances prescribed. There is also a review board responsible for monitoring and reporting on the provision of VAD services.

While some might argue the stakes involved (death of the patient) justify an extreme level of care in administering and regulating VAD, there are many other areas of healthcare where patient decisions are capable—or even highly likely—to lead to their death, that are not subject to this level of scrutiny. These include choosing surgical or pharmaceutical treatment options that are risky or predicted to have a low likelihood of success or refusing lifesaving interventions. This is not to imply these situations are taken lightly, or that careful documentation is not also required, but they do not involve the six levels of process seen in VAD. The patient’s autonomy and right to determine their care is the same, and the ultimate outcome of patient death will also often be the same. What appears to be different is the role of the physician: the patient may die in all the above scenarios, but it is only in VAD that the *aim* of the intervention is the death of the patient, and thus the *intention* of the physician is to bring about this outcome. Death is no longer the *foreseen but unintended side-effect* of relieving pain that we have relied on for so long to justify a physician’s actions in this regard. I argue that the DDE has been connected to the provision of euthanasia for so long that its assumptions and restrictions can be felt in this new VAD legislation.

To explore the legacy of DDE rhetoric over the provision of VAD under the *Voluntary Assisted Dying Act 2017 (Vic)*, a semantical content analysis was conducted on the text of the legislation. This included the entirety of the authorized version No. 005 (128 pages), excluding the six-page table of contents, the eight form templates appended to the document (totalling thirty pages), a one-page placeholder for repealed headings, and the endnotes (totalling six pages). The template forms were excluded as they substantively repeat the content already in the document, although it is noteworthy such templates are not common in legislative documents of this nature. The use of content analysis methodologies for exploring legislation has been recently defended by legal scholars (Salehijam 2018; Brook 2022). As the legislation is necessarily repetitive in parts, a simple word frequency calculation using NVivo software was deemed insufficient to capture the relevant data, so coding was done manually to preserve the context. Referencing Janis’ 1965 methodology, in their book on qualitative research methods, Stewart, Shamdasani, and Rook (2007) note semantical content analysis can involve the following:

1. Designations analysis: focused on the frequency with which items appear in the textual sample
2. Attribution analysis: focused on the frequency “certain characterisations or descriptors are used”
3. Assertions analysis: focused on the frequency with which items are “characterised in a particular way,” e.g., is a combination of the two above methods (Stewart, Shamdasani, and Rook, 2007, 119).

The present analysis engages the third method, with the main coded items being the person seeking VAD, the other people involved in the process, and the request for VAD itself. Manual coding was completed by repeated review of the whole text of the legislation and marking characterizations and descriptors relevant to the three main items. Words with particular relevance to bioethics were also highlighted and counted (e.g., autonomy, respect, consent, etc.) although as the genre of writing was legal, these key terms appeared infrequently.

## 4: Results of Semantical Content Analysis

The declared aim of the Act is to “provide for and regulate access to” VAD (VAD Act, Part 1, subsection 1a). This seems to be supported by the fact the words “access/ing” appear eighty-one times in the target eighty-five pages (excluding those instances appearing in page headers), and variants of “eligible/eligibility/ineligible” appear fifty-three times. Of the latter, forty-six instances are in direct reference to the person seeking VAD, and the others refer to eligibility for witnesses and interpreters involved in the process.

### 4a: Characterization of the Person Seeking VAD

There is a preoccupation in the wording of the legislation with interrogating the capacities of the person seeking VAD. The need for them to demonstrate understanding is specifically mentioned ten times (“understand/ing”), possessing “decision-making capacity” is a requirement noted twenty-one times, and the characterization of their decisions are that they must be made “freely” (2), “voluntarily” (8) and “without coercion” (6). There are also nine distinct references to the person being able to change their mind at any stage, with no obligation to continue the process. Again, one could argue that this level of caution is warranted due to the seriousness of the request being made, however, most of these requirements are already well covered in other relevant legislation (e.g., assessing decision-making capacity) and it is difficult to see how the redundancies within this document are necessary in isolation, let alone in concert with existing patient protections. In contrast to such repetition, the “quality of life” of patients at the end of life is only referenced once in the VAD Act, carrying a duty to provide “quality care to minimise the person’s suffering and maximise the person’s quality of life” (VAD Act, Part 1, subsection 5(1)d). Providing VAD or information about VAD are explicitly excluded from these duties. The word “consent” also only appears once in the document, in reference to gaining the person’s consent before telling their family members about the VAD plan and process (note, such a disclosure would also be covered by relevant sections of the *Health Records Act 2001* where consent processes are mentioned fifty-nine times). Three of the uses of “support/ed” refer to the person seeking VAD being supported in their decision-making, and a further instance refers to promoting the therapeutic relationship with their medical practitioner. “Autonomy” appears once, with a general statement that it should be respected.

### 4b: Characterization of the Request for VAD

Following from the above, there are also various requirements for the format of the VAD request. The term “request” appears 148 times in the target pages (271 times in the document as a whole), and that the request must come from the person seeking VAD “personally” is noted four times. It is also mentioned once that the request must be “clear and unambiguous” but thirteen times that it must be judged to be “enduring.”

### 4c: Characterization of Witnesses, Interpreters, and the Contact Person

It is noted in the legislation that witnesses and interpreters cannot be beneficiaries in the will of the person seeking VAD or stand to financially benefit from their death, cannot sign as proxies for the person on forms they are involved in, and cannot own the healthcare facility providing care. Only one of the two required witnesses can be a family member, and an interpreter must not be related to the person or be directly involved in providing care. There are also minimum levels of training expected of an eligible interpreter. Descriptors and characterizations of the contact person are mostly absent throughout the document, with most references to this individual just focusing on the technical aspects of their role.

### 4d: Characterizations of “Every Person” (and Similar Global Terms)

The legislation opens with some general principles which declare all human lives have “equal value,” that every person’s values, culture and beliefs are worthy of respect, and that every individual should be encouraged to discuss their end-of-life preferences. The Act also lists offenses that any person can commit, including using “dishonesty” (2), “undue influence” (2) or attempting to “induce” (4) someone to request or complete VAD.

### 4e: Characterizations of Medical Practitioners

By far the most interesting case in the legislation is how medical practitioners are depicted. They must be reminded fifteen times not to bring up the topic of VAD with people they are treating (“initiate/initiating” and “suggest/ing”) and twice that any breach of the Act, or failure to report a suspected breach, constitutes “unprofessional conduct.” They are also told three times that they can refuse to continue the process at any stage and once each that it is part of their role to assess “coercion” and possible “abuse.” Minimum qualifications are repeated six times and the need for specialized training in VAD thirteen times. They are also reminded three times that they can only administer the VAD substance if the person is “physically incapable” of self-administration. Of particular interest are Sections 7 and 8 of the Act. Section 7 outlines the rights of medical practitioners with a conscientious objection to VAD, which include refusing to provide any information or be involved at all in the process:

**7 Conscientious objection of registered health**

** practitioners**

A registered health practitioner who has a

conscientious objection to voluntary assisted

dying has the right to refuse to do any of the

following—

- to provide information about voluntary
  assisted dying;
- to participate in the request and assessment
  process; (VAD Act, Part 1, subsection 7).

As will be demonstrated in the next section, this is not the standard method of dealing with conscientious objection for procedures that have particular moral valence for some practitioners. Instead of a duty to refer, for example, the above gives the practitioner the right to refuse not only provision of the service themselves but also access to safe and accurate information about the intervention.

Regarding Section 8, Moore, Hempton, and Kendal (2020) have argued elsewhere that this section represents an unprecedented “gag clause” in Australian health legislation, as it forbids a medical practitioner from initiating discussion of a legally available intervention with an end-of-life patient. This puts the onus on patients to familiarize themselves with the law pertaining to VAD and explicitly request this from their medical practitioner. There are no comparable demands made of patients seeking other forms of healthcare, as the expectation is they can trust their physician to provide information that might be relevant to their situation. The text of this section is as follows:

**8 Voluntary assisted dying must not be initiated by**

** registered health practitioner**

1. A registered health practitioner who provides
  health services or professional care services to a
  person must not, in the course of providing those
  services to the person—

- initiate discussion with that person that is in
  substance about voluntary assisted dying; or
- in substance, suggest voluntary assisted
  dying to that person (VAD Act, Part 1, subsection 8).

The next section will demonstrate what is unique about the wording of both sections, and the characterization of healthcare providers more broadly in this Act.

### 4f: Comparison to the Abortion Law Reform Act 2008 (Vic)

Like euthanasia, abortion is a medical practice that elicits strong views in the community. While there are various ways in which it cannot be directly compared, the *Abortion Law Reform Act 2008 (Vic)* nevertheless provides a useful contrast for some of the issues identified above (*Abortion Law Reform Act 2008 (Vic),* Victorian Government, 2012, henceforth Abortion Act). One obvious difference is the length of the document, twelve pages in total, with only six of these covering the legislation itself (compared to 128 and eighty-five for the VAD Act, respectively). There are no specific forms included or any minimum qualifications listed for the medical practitioners involved (although it is a stated offense to perform an abortion if “unqualified”). The word “request” appears only once, and there are no descriptors included, e.g., unlike in the VAD Act, the terms “induce,” “coerce,” “voluntarily,” “freely,” “enduring,” “clear,” “unambiguous” or any similar words do not appear at all. Likewise, “decision-making capacity” and “understanding” for the woman seeking the termination of pregnancy are not specifically mentioned, neither are the terms “value/s,” “eligible/eligibility/ineligible,” “support/ed,” “discussion,” “initiate/initiating,” or “suggest/ing” present in the legislation. There is no reminder that the woman can change her mind at any time in the process. This in no way suggests medical practitioners do not have a duty to ensure informed consent, decision-making capacity, lack of coercion etc., regarding the decision to have an abortion, merely that all these duties are already covered in other legislation and physicians are trusted to apply these appropriately in the circumstance.

Another interesting difference is the phrasing of the conscientious objection section, an excerpt of which is included below:

**8 Obligations of registered health practitioner who has**

**conscientious objection**

1. If a woman requests a registered health
  practitioner to advise on a proposed abortion, or to
  perform, direct, authorise or supervise an abortion
  for that woman, and the practitioner has a
  conscientious objection to abortion, the
  practitioner must—

- inform the woman that the practitioner has a
  conscientious objection to abortion; and
- refer the woman to another registered health
  practitioner in the same regulated health
  profession who the practitioner knows does
  not have a conscientious objection to
  abortion (Abortion Act, subsection 8).

This is a far cry from being allowed to deny access even to *information* regarding VAD. Further, there is nothing comparable to the VAD Act’s Section 8 gag clause present in this, or any other, health legislation in Australia.

The same powerful lobbying groups opposing the passage of this reform were agitating to prevent the legalization of VAD in Victoria as well. So, the question becomes how did such pressure lead to the development of such an unwieldy piece of legislation in the one instance but not the other? I argue one explanation is rather than relying on the DDE, prior to the decriminalization of abortion in Victoria, therapeutically indicated abortion was covered by the Menhennitt Ruling (Supreme Court, 1969) (Cica 1998). This stated that termination of pregnancy may be lawful if necessary to preserve the physical and/or mental health of the pregnant woman, listing no specific qualifications for those involved in the process or any limitation on gestational age of the fetus. Instead of focusing on *intentions*, the Menhennitt Ruling requires that the person performing the abortion have an “honest and reasonable belief” the termination is necessary and proportionate in the circumstances, while the *Abortion Law Reform Act 2008 (Vic)* specifies the medical practitioner need make no judgment at all before twenty-four weeks’ gestation and after this point must, alongside a second medical practitioner, “reasonably believe” the termination is “appropriate in all the circumstances” (Abortion Act, Part 2, subsection 5). In the first instance, an honest belief may be as difficult to prove as an intention, but a *reasonable belief* can be objectively determined. This standard appears to alleviate the distrust of medical practitioners implied in the VAD Act, where they cannot even be allowed to “initiate discussion” of VAD, lest they unwittingly coerce the patient to choose death.

## 5: Abusing the Doctrine of Double Effect through “Purifying the Intention”

One of the major concerns for opponents of the DDE is the risk of abuse through “purifying the intention” (Pascal 1994). According to C. E. Kendall (2000), while the DDE provides a “useful framework” for determining action, it is also “open to abuse as it depends heavily on the integrity and honesty of the person applying it, and intent is very difficult to prove” (205). The basis of this objection therefore focuses on the fact that it is not necessarily possible to ascertain the true intentions of another person, and thus the DDE may be used as a means of justifying actions that are otherwise impermissible. In the area of medical ethics, Len Doyal (1999) claims that according to the DDE, any treatment option can be pursued no matter what the potential side-effects, as long as the doctor involved can remain “psychologically disciplined” in their professed intent of bringing about a good outcome (1432). However, it is important to remember that according to the four criteria outlined in the DDE, it is never acceptable to engage in an action which is not considered morally permissible independent of its consequences, and also that in any risk-benefit calculation the intended good outcome must outweigh the risks of harm caused by the unintended side-effect. These limitations already serve to regulate the use of the DDE to justify actions that can reasonably be expected to yield both positive and negative effects.

Blaise Pascal (1994) argues that according to the DDE, the intention behind a morally unacceptable act need only be “redirected” toward some permissible goal, for that action to be defended and its agent absolved from guilt (265). This implies that such a technique could hypothetically render any action permissible, whether the purification of intention is genuine or not. However, while it is certainly true that we can never be sure whether an agent truly has the morally acceptable intention they claim when defending their actions, I would argue that this is a problem that is not unique to the DDE but rather plagues many moral theories. Kant, for example, was preoccupied with establishing whether an agent acted out of inclination or duty, and thus Kantian ethics is as reliant on subjective assumptions as the DDE. Similarly, how can we be sure that a utilitarian acts with a genuine belief that their action will produce the greatest utility? Using the classical trolley example, the utilitarian may be able to defend their decision to sacrifice one life to save five, however, what if those five were the agent’s own children? Would we not then become suspicious that their true goal was not maximizing utility so much as protecting their own personal interests? Clearly, it is as difficult to determine true motivation in these cases, as it is any concept of intention with regards to the DDE. I argue it is therefore unreasonable to reject the DDE as a moral theory *on the grounds* that an agent may be able to “hide” their true intention, since it is not possible for competing theories to objectively validate an agent’s action either, whether in the light of duty, utility, or any other governing principle.

There is also a difference between evaluating an act and its consequences and determining whether an agent acted with an acceptable intention when engaging in that activity (Gillon, 1986, 193). I believe it is significant that the DDE does not attempt to identify moral and immoral actions but rather demands that any action subjected to evaluation already be considered morally permissible independent of its consequences. William Fitzpatrick (2003) claims the DDE therefore does not attempt to evaluate actions or their consequences but rather evaluates the moral character of the agent involved on the grounds of their intention (318). In this way “purifying the intention” may provide the illusion of an agent behaving morally, however, it cannot make a wrong action right. As James Rachels (1986) states: “intention is relevant, not to determining the rightness of actions, but to assessing the character of the people who act” (95). Pascal (1994) claims that through the DDE it is possible to “correct the viciousness of the means by the purity of the ends,” however, according to the above criteria this objection is invalid (265).

What is important when considering the legacy of the DDE on VAD, is the “morally permissible” act was never the causing of death but the alleviation of pain. However, death is not the “foreseen but unintended” side effect of VAD but the primary method of achieving the goal of ending irremediable suffering. The medical practitioner involved does not hope the patient continues to live but with less discomfort, merely recognizing this palliation carries with it the unfortunate side-effect of a shorter life expectancy. The VAD substance prescribed is intended to cause death and prescribed at a dose expected to achieve this outcome. But having historically established that hastening the death of end-of-life patients was only acceptable if the physician did not *intend* this, it then becomes necessary to remove their agency in the VAD process altogether. How else can we be sure this physician doesn’t secretly enjoy slaughtering their patients? Like in our vaccination example above, even if the outcome is the same, the moral valence here is significant. Since a VAD request and a VAD substance are explicitly intended to cause death, and our historical tolerance of medical practitioners being involved in hastening the death of suffering patients explicitly prohibited such an intention, it follows that they must be gagged, laden with administrative burdens, and prohibited from administering the VAD substance itself except under the direst of circumstances where the patient is incapacitated. Even if we try to rehabilitate the DDE to establish intentionally causing death in the pursuit of alleviating suffering as a morally permissible goal in its own right, the problem with elucidating the genuine motivations of the physician remain. The only way to be confident they are not merely “purifying their intentions” when suggesting VAD to a suffering patient, is to make it illegal for them to suggest it at all. This is different to the abortion example, where testing the reasonableness of a stated belief is more plausible. As the Abortion Act comparison demonstrates, the desires and agency of the medical practitioner are not typically given such prominence that they must be excluded from conversing openly with their patients *just in case* they are doing so with impure (coercive) intentions.

Similarly, while there are compelling reasons to prevent a financial beneficiary from influencing a person’s end-of-life choices, there are already protections in place to avoid abuse here. The simplicity of the *Abortion Law Reform Act 2008 (Vic)* is related to the fact many aspects involved in the process are already covered in other legislation. But this is no less the case for VAD, and yet many pages of this Act are devoted to describing convoluted processes of avoiding potential coercion. In ordinary treatment decisions it is not only permitted but *expected*, and often *legally required*, that a treating physician outline all the relevant treatment options for their patient. While this is meant to be done in a non-coercive fashion that maximizes patient autonomy, the concept of “nudging” patients to choose the options physicians’ deem most appropriate is widely discussed (Cohen 2013; Kiener 2021). Many such treatment decisions have real and immediate impact on the patient’s survival chances and long-term well-being. Yet we routinely trust medical practitioners, family members (including financial beneficiaries) and others to discuss these options with patients. Other, even potentially fatal, interventions can also be consented for by a Medical Treatment Decision-Maker on behalf of a patient lacking decision-making capacity, but again this is absolutely prohibited in the case of VAD (Victorian Government 2016). The DDE defence for opiate-based pain relief that will hasten death is still available, so euthanasia is in effect still legal in these cases. In short, we can still bring about the death of a patient without their clear, unambiguous, enduring, free, and voluntary request, just not *intentionally* or *efficiently*. The result is an inconsistent and discriminatory coverage of end-of-life care options for different populations: refusal and removal of life-sustaining interventions is permitted (either at the patient’s request or due to a judgment of medical futility), unintentional hastening of death via the DDE defence remains, but for those patients who do not fit in the above categories, VAD has only been added for the very limited number both eligible and personally knowledgeable of the option.

The more vulnerable the patient, the less likely they stand to benefit from the legalization of VAD as an end-of-life option. Patients with poor health literacy and who do not speak English as a first language face particular access challenges, while those with significant cognitive impairments are likely to be disqualified wholesale (Moore, Hempton, and Kendal 2020, 68). That this isn’t recognized as a form of discrimination rests on the assumption that VAD is inherently undesirable or does not genuinely constitute a treatment option one may fairly demand. Courtney Hempton (2021) also notes that VAD is not established as a “right” in the legislation and thus universal access is not provided for (3577). In fact, it seems to be treated as such a unique evil to the patient that they must be shielded even from the knowledge of its existence, wherever possible. That self-administration of the VAD substance is the default option, also characterizes VAD as something other than healthcare, which one would reasonably expect to be delivered by a healthcare practitioner. Again, this bears a resemblance to another traditional objection to the DDE, the issue of “closeness.”

## 6: Issues of “Closeness” in Determining Intention

This second common objection to the DDE is concerned with how closely related the bad outcome can be to the good outcome, before it must be said that the agent cannot possibly intend the good without also intending the bad. This is explored by Philippa Foot (1967) in a scenario dubbed the “Explorers,” in which it must be decided whether or not it is permissible to blow up a “fat man” who is stuck in a cave opening, effectively trapping several explorers inside. Foot argues that the intention of blowing up the man to escape the cave is inextricably linked to the death of the fat man and that the explorers therefore cannot truly argue that they do not intend to kill the man when they set the dynamite. Even if the explorers claim their intention was merely to reduce the size of the man by blowing him into bits, Foot argues that this is too “close” to intending the death of the man to be of any morally significant difference. Thus, according to Foot “anything very close to what we are literally aiming at counts as if part of our aim,” and thus the boundary between the foreseen and the intended starts to blur (Foot 1967). This objection is also supported by Elizabeth Anscombe (1970), who claims, “it is nonsense to pretend you do not intend to do what is the means you take to your chosen end” (51).

Using this logic, the doctor who inoculates the children with the intention of preventing disease cannot then argue that they did not also intend to puncture the skin and cause some inevitable pain, since the desired immunity and the pain of the injection are very closely linked. In this way, the doctor who causes pain as a side-effect seems to be held in equal moral standing as the malicious doctor who intends to hurt children, as the relationship between the good and the bad outcomes is so close as to arguably make intention irrelevant. However, I agree with Sarah Edwards (2001), that no matter how close two outcomes become, it is still possible to make a “morally independent” decision to intend one outcome while only foreseeing that the other is “chronologically or logically sequential” (391). Michael Bratman (1987) claims this distinction can be evidenced by a “commitment” to one end, which will dictate the specific plan adopted to achieve that end, as opposed to the *allowing* of certain other outcomes, which will have no significant influence on the choice of means applied, as they are not intended results but mere side-effects (140). With regards to the vaccination example, there may well be practical evidence that supports the genuineness of the first doctor’s intention, such as the adoption of a pain-free inoculation technique once it is proven to be as efficacious as the traditional injection method. This suggests that there can be proof, not only of the agent’s true intention but also that they genuinely do not *intend* the means to their end, if when provided with an alternative method they remain committed only to the desired good outcome and not the negative side-effect produced by their prior technique.

However, VAD is not like those cases where a physician prescribes pain relief that also hastens death, where at least some separation of the substance and death is maintained. We cannot distance the goal of ending intolerable suffering from bringing about the death of the patient, as the latter is the direct method of achieving the former. And according to the familiar rhetoric of the DDE, this would not be sanctioned. I argue that through the historical application of the DDE, we have grown accustomed to considering a medically induced death as only being acceptable when it is unintentional (especially from the physician’s perspective), which has left the *Voluntary Assisted Dying Act 2017 (Vic)* susceptible to socio-political influences intended to make it virtually unusable. Moreover, it assumes a position of suspicion and distrust of physicians that is not in keeping with cultural norms regarding the doctor–patient relationship. That “quality of life” is set up in opposition to VAD, also fails to recognize that a good death is part of a good life.

## 7: Conclusion: Re-conceptualizing VAD Outside of the Doctrine of Double Effect

How then should we classify VAD? Is bringing about death a legitimate method of reducing suffering, or is death a different category of thing altogether? Since death and relieving suffering are too closely linked in VAD to separate the “evil” method from the “pure” aim, I argue we should look to re-conceptualizing death in this case as a positive and desirable outcome for the person seeking VAD. Abandoning the belief that a physician cannot ethically *intend* to cause death will also alleviate some of the concerns about the impossibility of proving an agent’s intentions. Treating VAD consistently with other end-of-life interventions will open up more equitable distribution through removing the Section 8 gag clause and promoting open discussion about end-of-life preferences, including among patients with poor health literacy and other disadvantages. This will also avoid creating a precedent where religious or political influences can interfere with the norm of truth-telling in healthcare, which is aligned with patient expectations that they can trust their healthcare provider to act in their best interests and provide the specialist knowledge required for patients to make informed treatment decisions. In short, moving on from the rhetoric of the DDE will allow the benefits of VAD to be conferred to a wider population, reduce administrative burdens for healthcare practitioners and facilities, and promote a broader conversation about “quality of death” as an intrinsic component of “quality of life.”

## Data Availability

The text used in analysis is from the Victorian State legislation and thus publicly available (sources in reference list).
